# Theophylline-Based KMUP-1 Improves Steatohepatitis via MMP-9/IL-10 and Lipolysis via HSL/p-HSL in Obese Mice

**DOI:** 10.3390/ijms17081345

**Published:** 2016-08-17

**Authors:** Bin-Nan Wu, Kung-Kai Kuo, Yu-Hsun Chen, Chain-Ting Chang, Hung-Tu Huang, Chee-Yin Chai, Zen-Kong Dai, Ing-Jun Chen

**Affiliations:** 1 Department of Pharmacology, Graduate Institute of Medicine, College of Medicine, Lipid Science and Aging Research Center, Kaohsiung Medical University, Kaohsiung 807, Taiwan; binnan@kmu.edu.tw (B.-N.W.); sicaja9999@hotmail.com (Y.-H.C.); a99443@hotmail.com (C.-T.C.); 2 Division of Hepatobiliopancreatic Surgery, Kaohsiung Medical University Hospital, Kaohsiung Medical University, Kaohsiung 807, Taiwan; kkkuo@kmu.edu.tw; 3 Department of Anatomy, School of Medicine, Kaohsiung Medical University, Kaohsiung 807, Taiwan; hungtu@kmu.edu.tw; 4 Department of Pathology, School of Medicine, Kaohsiung Medical University Hospital, Kaohsiung Medical University, Kaohsiung 807, Taiwan; d750147@kmu.edu.tw; 5 Department of Pediatrics, Division of Pediatric Pulmonology and Cardiology, Kaohsiung Medical University Hospital, Kaohsiung Medical University, Kaohsiung 807, Taiwan; zenkong@kmu.edu.tw; 6 Department of Medical Education and Research, Pingtung Christian Hospital, Pingtung 900, Taiwan

**Keywords:** adipose triglyceride lipase, fatty liver, hormone sensitive lipase, M1/M2 macrophage, matrix metallopeptidase 9, tumor necrosis factor α

## Abstract

KMUP-1 (7-[2-[4-(2-chlorobenzene)piperazinyl]ethyl]-1,3-dimethylxanthine) has been reported to cause hepatic fat loss. However, the action mechanisms of KMUP-1 in obesity-induced steatohepatitis remains unclear. This study elucidated the steatohepatitis via matrix metallopeptidase 9 (MMP-9) and tumor necrosis factor α (TNFα), and related lipolysis via hormone sensitive lipase (HSL) and adipose triglyceride lipase (ATGL) by KMUP-1. KMUP-1 on steatohepatitis-associated HSL/p-HSL/ATGL/MMP-9/TNFα/interleukin-10 (IL-10) and infiltration of M1/M2 macrophages in obese mice were examined. KMUP-1 was administered by oral gavage from weeks 1–14 in high-fat diet (HFD)-supplemented C57BL/6J male mice (protection group) and from weeks 8–14, for 6 weeks, in HFD-induced obese mice (treatment group). Immunohistochemistry (IHC) and hematoxylin and eosin (H&E) staining of tissues, oil globules number and size, infiltration and switching of M1/M2 macrophages were measured to determine the effects on livers. IL-10 and MMP-9 proteins were explored to determine the effects of KMUP-1 on M1/M2 macrophage polarization in HFD-induced steatohepatitis. Long-term administration of KMUP-1 reversed HFD-fed mice increased in body weight, sGOT/sGPT, triglyceride (TG) and glucose. Additionally, KMUP-1 decreased MMP-9 and reactive oxygen species (ROS), and increased HSL/p-HSL and IL-10 in HFD mice livers. In conclusion, KMUP-1, a phosphodiesterase inhibitor (PDEI), was shown to reduce lipid accumulation in liver tissues, suggesting that it could be able to prevent or treat steatohepatitis induced by HFD.

## 1. Introduction

Studies of theophylline-based KMUP-1 (7-[2-[4-(2-chlorobenzene)piperazinyl]ethyl]-1,3-dimethylxanthine) have shifted to lipid metabolism and obesity [1] in addition to cardiovascular and neuronal systems [2,3,4]. KMUP-1 has been demonstrated as a phosphodiesterase inhibitor (PDEI) [5,6] and it also proved to reduce inflammation and hyperalgesia in a bilateral chronic constriction injury model by suppressing p38 mitogen-activated protein kinase (p38 MAPK) and NFκB activation [3]. Moreover, KMUP-1 caused hepatic fat loss by increasing protein kinase A (PKA) and protein kinase G (PKG), the cyclic nucleotide dependent protein kinases, resulted from cAMP and cGMP activation [1]. Enhanced PKA or PKG can phosphorylate perilipin on oil globules, resulting in lipolysis by activation of hormone sensitive lipase (HSL)/phosphorylated HSL (p-HSL) and adipose triglyceride lipase (ATGL) [7].

Recently, PDE5 inhibitors-mediated cGMP/PKG accumulations have been approved for the therapeutic uses of erectile dysfunction and pulmonary hypertension [1,5,6], but their effects on obesity are not extensively described. A famous PDE5 inhibitor sildenafil was shown to increase cGMP/PKG in lipid metabolism, which leads to adipogenesis or lipolysis [8,9,10]. Previous reports also showed that inhibition of phosphodiesterases (PDEs) lead to activation of HSL/p-HSL and ATGL, promoting lipolysis of adipocytes [8,9,11]. Those reports inspire us to reconsider the important role of our PDE inhibitor KMUP-1 in obesity. In this report, we observed that KMUP-1 affected high-fat diet (HFD)-induced hyperadiposity in livers through matrix metallopeptidase 9 (MMP-9) and reactive oxygen species (ROS) inhibition, and HSL/p-HSL and IL-10 stimulation, suggesting that it might be a potential candidate for strengthening the therapy of nonalcoholic fatty livers.

Hepatic steatosis induced by a HFD-mediated hyperadiposity in liver tissues, involving lipid accumulation combined with inflammation and setting the stage for further liver damage. Steatohepatitis is usually accompanied by oxidative stress via ROS after a long-term supplementation with HFD [12,13,14]. HFD-induced oxidative stress also activated stress pathways involving phosphorylation of p38 MAPK and ERK expression in hepatocytes [15]. In this study, we aimed to explore the pharmacotherapeutic agents that may be able to suppress ROS, inhibit pro-inflammatory cytokine TNFα and MMP-9, increase anti-inflammatory cytokine IL-10 and affect the infiltration of macrophages in liver tissues [16,17,18,19].

Immunostaining results display massive macrophage infiltration in HFD-induced liver inflammation in obese mice, characterized by macrophage types due to M1/M2 polarization [20]. We have measured mice body weight and biochemical parameters, and performed H&E and immunohistochemistry (IHC) staining from the number/diameter changes of oil globules and M1/M2 macrophage polarization in mice livers to investigate whether KMUP-1 reduced hepatic fat accumulation that is attributable to its anti-inflammatory and lipolytic activities.

## 2. Results

### 2.1. Body Weight Gain, sGOT, sGPT, Triglyceride (TG) and Glucose in Serum

**Figure 1:**
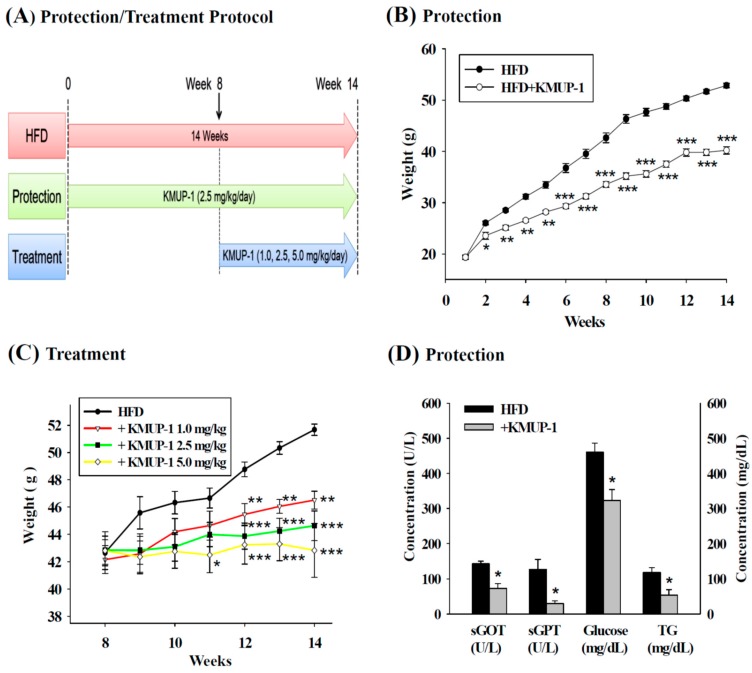
Effects of KMUP-1 on high-fat diet (HFD)-induced body weight, serum glutamic-oxaloacetic transaminase (sGOT), serum glutamic-pyruvic transaminase (sGPT), triglyceride (TG), and glucose in serum of mice treated with KMUP-1. (**A**) Protection/Treatment protocol in the mice model; (**B**) Oral administration of KMUP-1 (2.5 mg/kg/day for 14 weeks) prevented HFD-induced body weight increase; (**C**) Treatment with KMUP-1 (1, 2.5, 5 mg/kg/day for last 6 weeks) attenuated HFD-induced body weight gain from week 8 to week 14; (**D**) HFD-induced sGOT, sGPT, TG and glucose levels were reduced by KMUP-1. Data are means ± Standard error (S.E.), *n* = 6–8. * *p* < 0.05; ** *p* < 0.01; *** *p* < 0.001 versus HFD group.

Figure 1A depicts the protocol of protection and treatment groups in HFD-fed mice. Figure 1B shows the difference of body weight in the HFD group and the HFD + KMUP-1 group after oral administration of KMUP-1 (2.5 mg/kg/day) for 14 weeks. At the first week, the mean weekly body weight gain was not significantly different. From the second week, supplementation with oral KMUP-1 for 14 weeks in the protection group significantly prevented the increases of body weight. Treatment with KMUP-1 (1, 2.5, 5 mg/kg/day) for 6 weeks, from week 8 to week 14, reduced body weight in obese mice (Figure 1C). Figure 1D shows the lower serum glutamic-oxaloacetic transaminase (sGOT), serum glutamic-pyruvic transaminase (sGPT), TG and glucose levels in the serum of HFD experimental animals in the protection group.

### 2.2. Hematoxylin and Eosin (H&E) Staining of Livers

**Figure 2:**
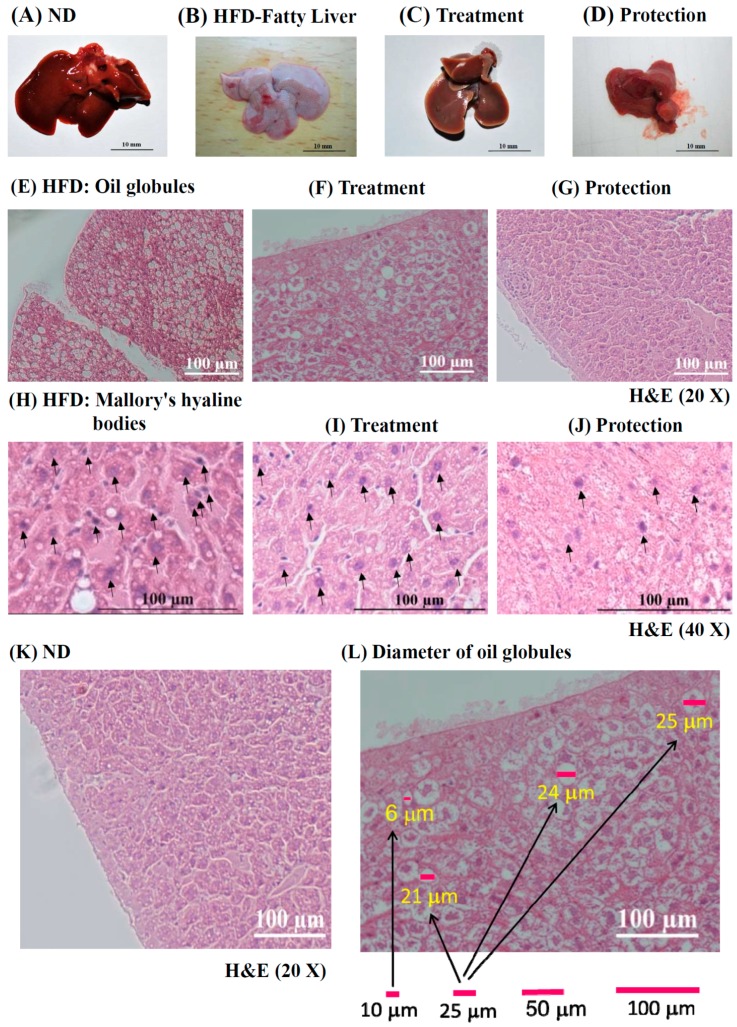
Morphology of livers protected or treated with KMUP-1 in HFD mice shown by H&E staining and the diameter of oil globules measured. (**A**) Normal morphology of livers in mice fed with normal chow diet (ND) for 14 weeks; (**B**) Mice fed with HFD for 14 weeks induced relevant fatty liver; (**C**,**D**) represent the Treatment and Protection of liver changes by oral gavage of KMUP-1 (2.5 mg/kg/day) for 14 weeks in HFD mice; (**E**) Excessive oil globules in HFD liver at 14 weeks; (**F**) Treatment and (**G**) protection of fatty livers by KMUP-1 for 6 weeks and 14 weeks, respectively; (**H**) Large amount of Mallory’s hyaline bodies (purple dots indicated by black arrow) were observed in the HFD group; (**I**) Obese animal treated with KMUP-1 (2.5 mg/kg/day) for 6 weeks decreased the worst pathologic changes at 14 weeks, i.e., the oil globules and Mallory’s hyaline bodies were attenuated at 14 weeks; and this response was more prominent in the (**J**) protection group; (**K**) shows H&E staining of the liver from normal chow diet (ND) mice as a control; (**L**) A representative example for measuring of hepatic oil globules from Figure 2F. The standardized diameters of 10, 25, 50 and 100 µm are depicted.

Figure 2A exhibits the normal gross morphology of mice livers fed with normal chow diet (ND) for 14 weeks. By contrast, Figure 2B shows the gross morphology of an inflammatory fatty liver fed with HFD for 14 weeks, which was fat tissues rich compared to the reddish-brown livers found in the treatment (Figure 2C) and protection group (Figure 2D) supplemented with KMUP-1 + HFD. Figure 2E shows the liver section of oil globules, also confirmed by Oil Red O staining, in the HFD group. Liver sections of mice treated with KMUP-1 (2.5 mg/kg/day, p.o.) for the last 6 weeks (treatment group) and 14 weeks (protection group) were shown in Figure 2F,G. Mice supplemented with HFD induced obviously oil globules and Mallory’s hyaline bodies (black arrow, Figure 2H). Oral KMUP-1 for the last 6 weeks reduced oil globules and Mallory’s hyaline bodies (Figure 2I). Notably, oil globules are dramatically decreased in the protection group (Figure 2J). Figure 2K displays H&E staining of the mice liver from normal chow (ND) diet as a negative control. The estimated diameter of oil globules from Figure 2F was 6, 21, 24 and 25 µm by using a free software ImageJ (Figure 2L).

### 2.3. Immunohistochemistry (IHC) Staining of TNFα/MMP-9/HSL/p-HSL/ATGL in Steatohepatitis

**Figure 3:**
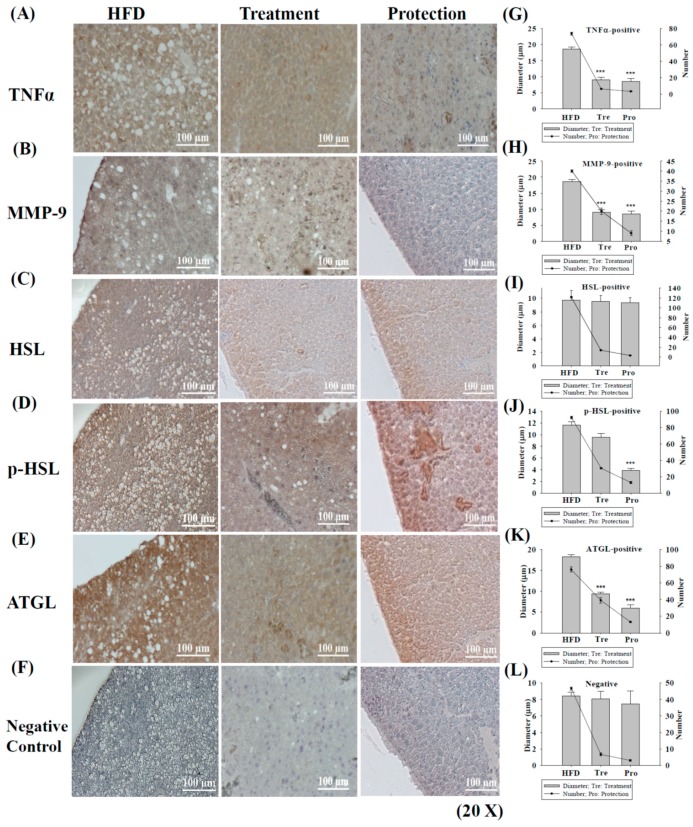
Immunohistochemistry (IHC) staining of tumor necrosis factor α (TNFα)/matrix metallopeptidase 9 (MMP-9), hormone sensitive lipase (HSL)/phosphorylated HSL (p-HSL) and adipose triglyceride lipase (ATGL) in HFD-induced liver steatosis and oil globules protected/treated with KMUP-1 for 14 weeks/6 weeks. HFD-induced fatty liver at 14 weeks implied that oil globules were rich in liver tissues. The expression of HFD-induced TNFα (**A**) and MMP-9 (**B**) in the treatment and protection groups; Treatment and/or protection with KMUP-1 sharply reduced the number and diameter of oil globules (**G**,**H**); HFD-induced the expression of HSL/p-HSL showed that KMUP-1 could affect the HSL protein (brown, **C**); and significantly enhanced the active form of HSL (p-HSL) in the protection group (deep brown, **D**) and matched data regarding the number and diameter of oil globules depicted in (**I**,**J**); ATGL expression is not affected by KMUP-1 in both treatment and protection groups (**E**); but markedly attenuated the number and diameter of oil globules (**K**); (**F**,**L**) negative control of protein expression, and the number and diameter of oil globules. Data are means ± S.E. of three independent experiments. *** *p* < 0.001 versus HFD group.

Figure 3 shows that oral administration of KMUP-1 (2.5 mg/kg/day) for 6 weeks (treatment group) and 14 weeks (protection group) only slightly affected TNFα expression (Figure 3A), but the number and diameter of oil globules significantly reduced. In Figure 3B, MMP-9 was nearly abolished in the protection group and oil globules markedly decreased as well, indicating the anti-inflammatory effect of KMUP-1 (Figure 3B,H).

In Figure 3C,D, the HSL/p-HSL is an intracellular enzyme of adipose tissue catalyzes the breakdown of stored TGs into glycerol and fatty acids (this process is called lipolysis), with the latter entering the circulation. The HSL is affected by KMUP-1 in the protection group and the number of oil globules was decreased, but not diameter (Figure 3I). In Figure 3D, KMUP-1 also enhanced the phosphorylated HSL (p-HSL, activated form of HSL) in the protection group, indicating the stimulation of lipolysis in oil globules, and the accompanied changes in number and diameter were reduced exactly (Figure 3J). These results support that KMUP-1 inhibition of steatohepatitis is more prominent in the protection group than the treatment group.

Likewise, adipose triglyceride lipase (ATGL) is another key enzyme involved in intracellular degradation of TGs in adipose tissues. The ATGL expression appears little affected by KMUP-1 in both treatment and protection groups (Figure 3E), but the number and diameter of oil globules were markedly diminished (Figure 3K). Even in the negative control, the number of oil globules in the HFD group also significantly decreased after KMUP-1 treatment and/or protection (Figure 3F,L).

### 2.4. IHC Staining of Type 1 or Type 2 Macrophages (M1 or M2) in Steatohepatitis

**Figure 4:**
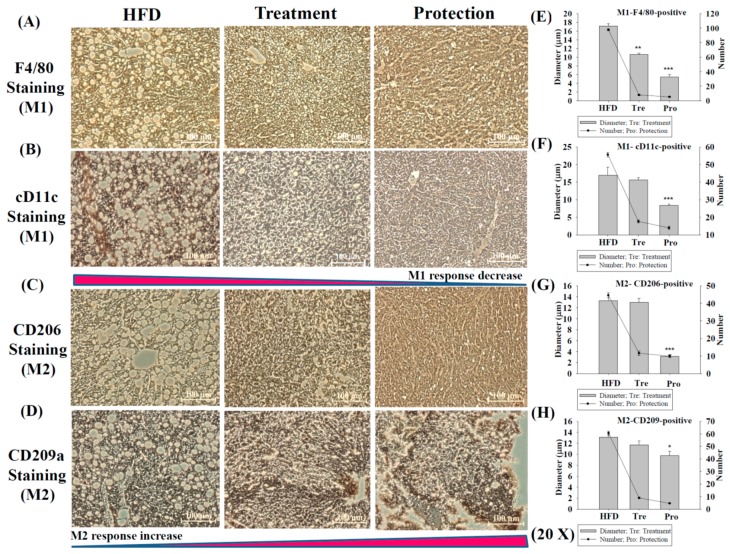
IHC staining of infiltrated M1/M2 macrophages and accumulated oil globules treated/protected with KMUP-1. KMUP-1 (2.5 mg/kg/day) administration for 6 weeks/14 weeks modulates the balance of infiltrated macrophages 1 (M1; **A**,**B**) and macrophages 2 (M2; **C**,**D**). KMUP-1 significantly affected the M1 (CD11c, dark brown)/M2 (CD209a, dark brown) macrophages polarization, but little affected the balance of M1 (F4/80, brown) and M2 (CD206, brown), in treatment and protection groups. All the accompanied oil globules were reduced by KMUP-1. The number and diameter changes of oil globules are in the average from M1/M2-positive cells (**E**–**H**). Data are means ± S.E. of three independent experiments. * *p* < 0.05; ** *p* < 0.01; *** *p* < 0.001 versus HFD group. Scale bar: 100 µm.

Figure 4 indicates the decreases in M1 (induce proinflammatory cytokines) and increases in M2 (decrease inflammation and promote tissue repair) macrophages by KMUP-1, staining with F4/80 and CD11c antibodies for the M1 type (Figure 4A,B), and with CD206 and CD209a antibodies for M2 type (Figure 4C,D). The number and diameter changes of oil globules in the treatment and protection groups were significantly different from the HFD group as shown in Figure 4E–H. The bidirectional arrow indicates the decreased M1 (CD11c) and/or increased M2 (CD209a) macrophage responses.

### 2.5. Expression of IL-10 and MMP-9 in HFD Livers

**Figure 5:**
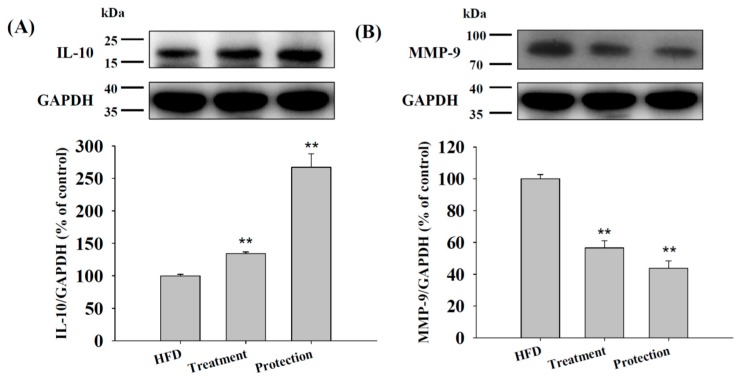
Expression of IL-10 and MMP-9 in mice livers treated with KMUP-1. Treatment/protection with KMUP-1 (2.5 mg/kg/day) for 6 weeks/14 weeks increased the expression of IL-10 (**A**) and decreased the expression of MMP-9 (**B**) in mice livers. The protein expression of IL-10 and MMP-9 was described in Materials and Methods. Data are means ± S.E. of six independent experiments. ** *p* < 0.01 versus HFD group.

Figure 5A shows that KMUP-1 increased IL-10 and would be related to the tendency of M2 type manifestations of macrophage. Additionally, Figure 5B shows that KMUP-1 decreased MMP-9, upregulated by proinflammatory mediators, and would be related to the tendency of M1 type manifestations of macrophage. Taken together with Figure 4 data, we suggested that KMUP-1 could influence the shift from M1 to M2 macrophages in HFD-induced mice livers.

### 2.6. Effects of Hyperadiposity on Hepatic Reactive Oxygen Species (ROS)

**Figure 6:**
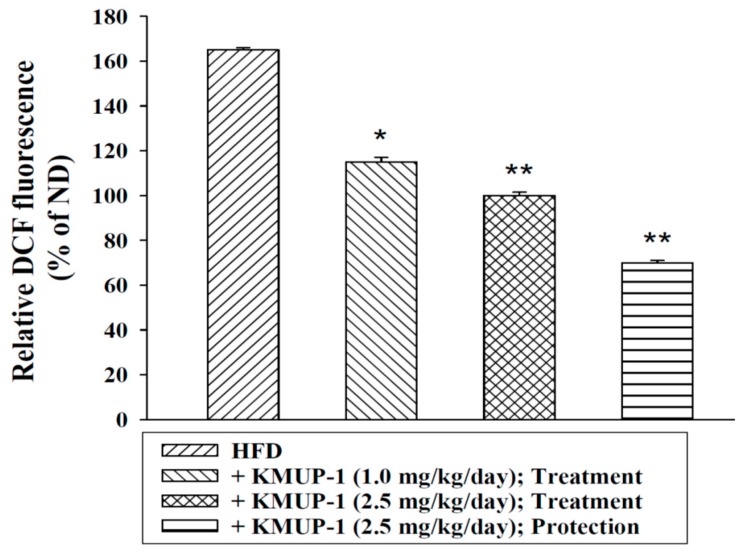
The levels of hepatic ROS was reduced by treating KMUP-1 (1, 2.5 mg/kg/day). HFD induced accumulation of ROS in livers. Protection/treatment with KMUP-1 for 6 weeks/14 weeks decreased the hepatic ROS. ROS was determined as described in Materials and Methods. Data are means ± S.E. of six independent experiments. * *p* < 0.05; ** *p* < 0.01 versus HFD group. ND: normal chow diet.

Hyperlipidemia increased the ROS of hepatic tissues detected by H2DCF-DA assay using fluorescence analysis (Figure 6). KMUP-1 reduced HFD-induced the increases of dichlorofluoroscence intensity in livers, suggesting that it could attenuate the levels of hepatic ROS.

## 3. Discussion

This study first provided a simple and reproducible method to measure and analyze the number/diameter of oil globules in mice liver using the digital image processing with the aid of ImageJ software, in comparison with previous investigations [21,22]. The oil globules in liver slices has been further confirmed by Oil Red O staining (data not shown). Oil Red O is widely used to validate the presence of fat or lipids in fresh and frozen tissues. On the other hand, sGOT and sGPT are used as two of biomarkers to measure routinely as a diagnosis of liver function. Suppression of these two biomarkers in mice by theophylline-based KMUP-1 is suggested that HFD-induced liver inflammation could be reduced by KMUP-1. Reduction of serum TG and glucose levels might positively correlate with lipids-associated metabolism syndrome and obesity-related insulin resistance in mice chronic inflammation [23].

Many hormones and drugs have been recognized to play a role in the modulation of lipid metabolism, and various hormones and drugs lead to lipolysis through diverse lipolytic pathways. PKA is involved in catecholamine-induced lipolysis, and PKG is responsible for lipolysis stimulated by atrial natriuretic peptide [8,10,24]. The most studied lipolytic pathway is the PKA pathway in adipocytes, in which catecholamines bind to β-adrenoreceptors and stimulate membrane-bound adenylate cyclases and accordingly raise the cAMP levels [10,25]. Elevated cAMP levels enhance PKA activity, leading to the phosphorylation and activation of HSL and lipid droplet-associated perilipin. Activated HSL and perilipin provoke the hydrolysis of TG stored in oil globules and the release of free fatty acids and glycerol from adipocytes [1,10,26]. In a previous report [1] we also confirmed that elevated cGMP/PKG in liver tissues potentially influences the lipid catabolism of hepatocytes by lipolysis of oil globules through HSL.

Interestingly, in our HFD-fed mice model, a relatively large amount of oil globules in the liver slice was mitigated in the treatment and protection groups in spite of some inflammatory and lipolytic proteins expression being little affected by KMUP-1. Oral administration of KMUP-1 resulted in a greater increase in the response of p-HSL than HSL in the protection group, indicating that partial HSL is transferred to the active form of p-HSL, which would be able to decrease the development of hepatic steatosis through stimulating lipolysis. TNFα, a proinflammatory cytokine, enhances hepatic fat deposition by affecting the liver lipogenetic metabolism involving sterol regulatory element binding protein-1c (SREBP-1c) [16,17,18]. It also plays a physiological role to stimulate basal lipolysis through a decrease in the lipid-binding protein, perilipin [18,26]. TNFα also downregulates ATGL in adipocytes [17]. Deficiency in liver ATGL causes progressive hepatic steatosis. In HFD-fed mice livers, the downregulation of ATGL via TNFα caused progressive steatohepatitis [13,14]. MMP-9 is recognized as a more intense mediator than TNFα in liver inflammation [21,22]. In this study, KMUP-1 increased IL-10 and decreased MMP-9 significantly, little affected TNFα, indicating its anti-inflammatory properties in hepatic steatosis. This result can be further confirmed that KMUP-1 reduced Mallory’s hyaline bodies, which is a key pathological feature in alcoholic and non-alcoholic steatohepatitis, in HFD-fed mice. Taken together, KMUP-1 improves steatohepatitis that is attributed to decrease MMP-9, increase IL-10, and stimulate lipolysis via HSL/p-HSL.

Infiltration of hepatic macrophages from blood-born monocytes has been found in inflammatory livers. Macrophages that encourage inflammation are called M1 macrophages, whereas those that decrease inflammation and encourage tissue repair are called M2 macrophages; the former can release TNFα and the later can release IL-10 [20,23]. The expression of M1 and M2 type macrophages was analyzed by IHC in HFD-fed mice livers [20,23]. Most of the F4/80-positive/cD11c-positive M1 macrophages and CD206-positive/CD209a-positive M2 macrophages in the liver tissues were clearly separated by IHC staining [23]. Figure 4 shows that hepatic cD11c staining (M1) and CD209a staining (M2) were decreased and increased, respectively, in the protection and treatment groups compared to the HFD group. The number/diameter of oil globules was reduced by KMUP-1 under the same conditions. Thus, we suggested that KMUP-1 can reduce the proinflammatory M1 macrophage phenotype, but enhance the anti-inflammatory M2 macrophage phenotype in mice livers. Additionally, KMUP-1 also can protect against M1 macrophage-derived ROS, and therefore it is suggested to be able to reduce ROS-related oxidative stress, inflammation and steatohepatitis.

## 4. Materials and Methods

### 4.1. Animals and Blood Sampling

C57BL/6J male mice (20–22 g) were fasted for 24 h and then changed to a HFD (Basal purified Diet W/60% energy from fat, Blue:58G9 Test Diet; St. Louis, MO, USA) to produce an obesity model. At 6 weeks of age, the mice were randomly divided into 5 groups, two control and three treatment groups. The control mice received HFD without KMUP-1 and the pretreatment group received oral KMUP-1 (2.5 mg/kg/day) by gavage for 14 weeks (protection group). The obese mouse treatment group was fed a HFD with oral KMUP-1 (1, 2.5, 5 mg/kg/day) from week 8 to week 14. All animals were separated in plastic cages for feeding and drinking [1]. All procedures and protocols were approved (IACUC 100172, 13 May 2013) by the Animal Care and Use Committee at Kaohsiung Medical University and complied with the Guide for the Care and Use of Laboratory Animals published by the US National Institutes of Health.

TG and glucose in mouse serum were measured by the same methods used in the clinic. In brief, mouse blood was obtained by cardiac puncture followed by centrifugation at 1000 rpm to separate serum, and freezing at −80 °C for biochemical analysis using a Hitachi Clinical Analyzer 7070 (Hitachi High-Technologies Co. Tokyo, Japan). Agents used in the assays were obtained from Sigma-Aldrich Chemical Co. (St. Louis, MO, USA). To measure hepatic protein expression, KMUP-1 was administered for 14 weeks or 6 weeks before the mice were sacrificed [1]. The livers were obtained after cardiac puncture on the last day of the experiments.

### 4.2. Measurement of Hepatic Oil Globules Diameter

The diameter of the scale bar in each image was standardized at 100 µm for measuring the specific diameter of oil globules in a whole liver slice observed by microscope, and analyzed with the aid of ImageJ 2.1.4.9 software. An increase in oil globules diameter and cell number indicated increasing liver steatosis, i.e., steatohepatitis. Oil globules in liver slices were observed with a Nikon Eclipse TE2000-S microscope (Tokyo, Japan). The oil globules were counted from a bigger size to a smaller one until the observation reached its limit. Too small or lysis oil globules in liver slice was excluded in the counting process. Considering the possible damage by alcohol and other organic solvents on cell membrane in staining procedures, the abnormal oil globules was also excluded.

### 4.3. Hematoxylin-Eosin (H&E) Staining of Liver Tissues

Mice livers were cut and soaked in formalin, dehydrated through graded alcohols and embedded in paraffin. Specimens of liver tissues fixed with formalin (4%) were embedded in paraffin for 1 h at 4 °C cut into 4-μm-thick sections from paraffin-embedded and de-paraffinized tissue blocks, immersed in xylene and rehydrated with graded alcohols and subjected to H&E staining before examination by light microscopy.

### 4.4. Immunohistochemistry (IHC) Staining of Liver Tissues and Macrophages

The staining of liver tissues was performed as previously described [1]. Briefly, mice livers were fixed in 10% formalin for 24 h and then embedded in paraffin. For IHC of hepatic TNFα, MMP-9, HSL/p-HSL and ATGL, antigen retrieval of deparaffinized sections was performed in Dako target retrieval solution, pH 9.0 in a vegetable steamer followed by quenching of endogenous peroxidase activity with 3% H_2_O_2_ in methanol. Sections were then incubated with specific primary antibodies overnight at 4 °C in a humidified chamber. The antibodies of HSL/p-HSL (Cell Signaling, Boston, MA, USA), MMP-9 (Abcam, Cambridge, UK), TNFα (Abcam) and ATGL (Cell Signaling) were used. The sections were then examined using a DAKO EnVision Detection System kit (DAKO, Carpinteria, CA, USA) and counterstained with hematoxylin. Images were obtained through a Nikon Eclipse TE2000-S microscope.

For the staining of macrophages infiltrated into liver tissues, F4/80 and CD11c (Abcam) were used to stain M1 macrophages, and CD206 and CD209a (Santa Cruz Biotechnology, Santa Cruz, CA, USA) were used to stain M2 type macrophages.

### 4.5. Western Blotting Analysis in Liver Tissues

To detect the expression of IL-10 and MMP-9 proteins, liver tissues were cut into small pieces, placed into buffer for protein extraction and centrifuged at 20,000× *g* for 30 min. The obtained protein extract was boiled to a ratio of 4:1 with sample buffer (Tris 100 mM, pH 6.8, glycerol 20%, SDS 4% and bromophenol blue 0.2%). Electrophoresis was performed using 10% SDS-polyacrylamide gel (1 h, 100 V, 40 mA, 20 µg protein) and then transferred to polyvinylidene difluoride (PVDF) membranes (Millipore, Temecula, CA, USA). The membrane was blocked with 5% milk in Tris-buffered saline with Tween 20 (TBS-T) for 1 h and thereafter incubated with specific protein antibody. After the secondary antibody was conjugated with horse radish peroxidase (HRP) (1:5000 dilutions in 5% milk) for 1 h, the signals on the membrane were identified using enhanced chemiluminescence (ECL)-plus luminal solution and exposed to X-ray film for autoradiography.

### 4.6. Measurement of Hepatic ROS

Hepatic ROS was measured using 2′-7′-dichlorofluorescein (H2DCF-DA, Molecular Probe, Waltham, MA, USA). Briefly, 10 μL of liver tissue extracts was diluted 100-fold with cold PBS and labelled with 5 μmol/L 2′-7′-dichlorofluorescein, and the mixture was incubated at 37 °C for 30 min. Fluorescence was measured at 485 nm excitation and 530 nm emission to determine the concentration of H_2_O_2_ [27].

### 4.7. Statistical Evaluation

The experimental results were expressed as means ± S.E. Statistical differences were determined by one-way analysis of variance (ANOVA) or repeated-measures ANOVA. When appropriate, a Tukey-Kramer pairwise comparison was used for post hoc analysis. A *p* value less than 0.05 was considered significant in all experiments.

## 5. Conclusions

Obesity, that is, the extravagant accumulation of adipose tissue, is associated with poor health outcomes due to several metabolic and cardiovascular diseases. Adipose tissue inflammation mediates the correlation between excessive body fat accumulation and several inflammatory complications [16]. In this study, we observed that KMUP-1 is able to decrease MMP-9, increase IL-10, and stimulate lipolysis via HSL/p-HSL. In conclusion, theophylline-based KMUP-1 protects and/or inhibits liver inflammation and fat accumulation, suggesting that it could be invaluable for the treatment or prophylaxis of obesity-driven steatohepatitis.
